# Viral Infections and Their Ability to Modulate Endoplasmic Reticulum Stress Response Pathways

**DOI:** 10.3390/v16101555

**Published:** 2024-09-30

**Authors:** Flávio Guimarães da Fonseca, Ângela Vieira Serufo, Thiago Lima Leão, Karine Lima Lourenço

**Affiliations:** 1 Laboratório de Virologia Básica e Aplicada, Departamento de Microbiologia, Instituto de Ciências Biológicas, Universidade Federal de Minas Gerais, Av. Pres. Antônio Carlos, 6627, Pampulha, Belo Horizonte 31270-901, MG, Brazil; fdafonseca@icb.ufmg.br (F.G.d.F.); thiagolleao@gmail.com (T.L.L.); 2 CT Terapias Avançacadas e Inovadoras, CTERAPIAS, Universidade Federal de Minas Gerais, Belo Horizonte 31270-901, MG, Brazil; vs.angela@gmail.com

**Keywords:** cell stress, endoplasmic reticulum stress, unfolded protein response, poxvirus

## Abstract

In eukaryotic cells, the endoplasmic reticulum is particularly important in post-translational modification of proteins before they are released extracellularly or sent to another endomembrane system. The correct three-dimensional folding of most proteins occurs in the ER lumen, which has an oxidative environment that is essential for the formation of disulfide bridges, which are important in maintaining protein structure. The ER is a versatile organelle that ensures the correct structure of proteins and is essential in the synthesis of lipids and sterols, in addition to offering support in the maintenance of intracellular calcium. Consequently, the cells needed to respond to demands caused by physiological conditions and pathological disturbances in the organelle homeostasis, leading to proper functioning of the cell or even programmed cell death. Disturbances to the ER function trigger a response to the accumulation of unfolded or misfolded proteins, known as the unfolded protein response. Such disturbances include abiotic stress, pharmacological agents, and intracellular pathogens, such as viruses. When misfolded proteins accumulate in the ER, they can undergo ubiquitination and proteasomal degradation through components of the ER-associated degradation system. Once a prolonged activity of the UPR pathway occurs, indicating that homeostasis cannot be reestablished, components of this pathway induce cell death by apoptosis. Here, we discuss how viruses have evolved ways to counteract UPR responses to maximize replication. This evolutionary viral ability is important to understand cell pathology and should be taken into account when designing therapeutic interventions and vaccines.

## 1. Impact Statement

Viruses and their hosts have been co-evolving for billions of years. As a result, cells have developed countless strategies to cope with the deleterious effects caused by virus infections. However, evolution works both ways, and the parasitic viruses have also evolved numerous counteractions to deal with cell strategies to eliminate them. The unfolded protein response pathways may have evolved as an antiviral strategy, as a way to deal with stressful massive protein production in the endoplasmic reticulum caused by replicating viruses, leading to cell death and infection elimination. Not surprisingly, virus have developed strategies to cope with UPR responses, blocking death signaling triggered by UPR sensors, and even taking advantage of pro-life signals that cells use to restore ER homeostasis. Here, we visit known strategies devised by viruses to counteract or benefit from cellular unfolded protein responses.

## 2. Introduction

Most proteins synthesized by eukaryotic cells are cytoplasmic. However, a significant percentage is destined for endo/exocytic pathways. To this end, these proteins have a signal sequence marking them for translocation from the cytoplasm to the endoplasmic reticulum (ER) lumen. Protein synthesis with the signal sequence occurs mainly on ribosomes attached to the ER membrane, and the signal sequence helps direct the ribosome to the organelle membrane [1,2]. The ER lumen is a unique environment that contains the highest concentration of Ca^2+^ ions within the cell due to the active transport of Ca^2+^ ions by calcium ATPases. When compared to the cytosol, the ER lumen is an oxidative environment, and that is essential for the formation of molecular bridges and disulfide bonds in maturing proteins, stabilizing the protein structure. Because proteins are translocated as unfolded polypeptide chains, the lumen ER contains a myriad of calcium-dependent chaperones, lectins, foldases, and glucosidases that assist in the correct folding and assembly of proteins, allowing the acquisition of functional and stable tertiary and quaternary structures. The ER is a multifunctional organelle that ensures the correct structure of proteins’ coils and has a key role in the synthesis of lipids, sterols, and maintenance of calcium intracellularly [3,4]. As a result of being such an instrumental organelle, the ER is sensitive to disturbances on cellular homeostasis that are triggered by different types of stress signals, which may be of endogenous or exogenous origin and include, for example, cell chemical damage, harmful genetic mutations, nutrient insufficiency, cell differentiation, and also infections by different pathogens [5,6,7]. In eukaryotic cells, accumulation of unfolded or misfolded proteins in the ER works as a danger signal detected by different resident sensors in the organelle’s membrane. Activation of such sensors triggers chemical cascades that culminate in the expression of chaperones and foldases, components of the degradation system associated with the ER (the ERAD), protein secretion, and increase in phospholipid synthesis. All these countermeasures contribute to the recovery of the ER functionality and cell homeostasis. The coordinated activation of the sensors constitutes a specific response to ER stress, a physiological condition called the malformed protein response or UPR [8,9,10,11,12]. The UPR can be found from simple to more complex organisms from yeasts to mammals [13]. Indeed, all eukaryotes share similar mechanisms for coping with ER stress, including the increased expression of chaperones that are responsible for the refolding of proteins, and proteases that degrade irreversibly unfolded proteins. In yeasts, for example, the increased levels of unfolded proteins can lead to the production of a specific transcription factor that triggers the increased expression of chaperones. All Metazoans also have an additional mechanism to deal with ER stress: the attenuation of translation by inhibiting the formation of a complex between the two ribosomal subunits [14]. Quite similarly, mammalian cells can increase chaperone levels regulated by transcription factors that are produced as a result of the activation of a protein homologous to the one found in the yeast UPR pathway (which was the first to be characterized). From an evolutionary perspective, as organisms gained complexity, there was also an increase in the UPR pack of tools, with the gain of a number of molecules affecting a great number of cellular mechanisms. Initially, the restoration of cellular homeostasis was linked to the transcriptional and translational regulation, processes well studied in mammals [13,15]. A paradox of the UPR pathway is that it leads to a response with simultaneous activation of cell survival and pro-apoptotic pathways [16,17,18].

The first discovery and best-characterized pro-apoptotic pathway is the production of the transcription factor CCAAT enhancer-binding protein homologous protein (CHOP) also known as growth arrest and DNA damage-inducible gene 153 (GADD153), which is regulated by all arms of the UPR pathway [8,9,19]. The mechanism by which CHOP leads to cell death is still unclear. However, this regulation occurs through the transcriptional and post-translational modification of various BCL-2 family members involved in the core mitochondrial apoptosis pathway. During prolonged ER stress, ATF4 promotes apoptosis by increasing the expression of the transcription factor C/EBP homologous protein and by elevating oxidative stress and protein synthesis levels [8,20]. CHOP also induces the transcription of ER oxidoreductases, such as ERO1a, making the lumen of the reticular cisternae a super-oxidizing environment that participates in cell death. CHOP protein production “fluctuates” over time, being robustly induced in the initial phase after exposure of cells to stress-inducing drugs, followed by a decline in CHOP levels, whereas chaperones remain upregulated. In contrast to pro-apoptotic activity, it has been suggested that CHOP activates the transcription of GADD34, which interacts with protein phosphatase I to catalyze the dephosphorylation of eIF2α, restoring protein synthesis and cellular homeostasis [21,22].

One of the main mechanisms of cellular adaptation to ER stress is the stable upregulation of the 78kDa glucose-regulated GRP78, a molecular chaperone residing in the ER lumen that is also known as binding protein (BiP) or heat shock 70 kDa protein 5 (HSPA5), as well as other proteins that are involved in relieving the folding stress of proteins in the ER, including GRP94, ERp57, calreticulin, calnexin PDI disulfide isomerase, and p58IPK. However, studies by Rutkowski et al. (2006) suggest that, in general, the determining factor for cell adaptation to ER stress is the composition of proteins, rather than large variations in differential gene expression [22]. Furthermore, these authors suggest that ATF4, CHOP, and GADD34 proteins form an integrated network capable of converting a linear stress gradient in a life-and-death binary signal [20,22,23]. Other pro-apoptotic events are initiated by the UPR pathway, including phosphorylation of c-Jun N-terminal kinase (JNK), cleavage of caspases (cysteine-dependent aspartate-specific proteases) specific to the ER, activation of p53, and loss of cellular calcium homeostasis [8,9,17,24,25]. In recent years, several new functions have been described for the UPR pathway, including important roles in the development and functioning of the immune system [26,27,28].

### 2.1. Endoplasmic Reticulum Stress Sensors

When the ER metabolic capacity is overloaded, the UPR pathway is activated. This activation may be the result of different physiological conditions such as differentiation and development of specialized cells with a high rate of protein secretion, such as pancreatic cells and B lymphocytes, altered metabolic conditions, mutation in coding genes for endo/exocytic pathway proteins, as well as infection by certain pathogens or inflammatory processes. Furthermore, experimental interventions are known to induce the UPR, including the inhibition of N-glycosylation in the endoplasmic reticulum, inhibition of transport of proteins from the ER to the Golgi, depletion of intracellular calcium stores, inhibition of the proteasome, or expression of mutated proteins that saturate the folding capacity of the endoplasmic reticulum [5,29].

**Figure 1:**
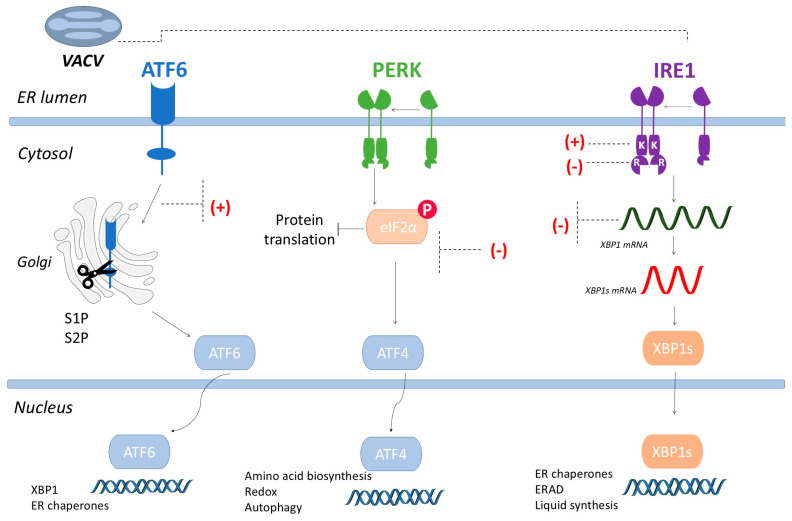
VACV infection and its impact on UPR pathway sensors. UPR track sensors detect misfolded proteins in the ER, resulting in the expression of target genes of the UPR pathway. There is a different signal transduction mechanism for each sensor in the UPR pathway: IRE1 through un-conventional mRNA splicing, ATF6 through regulated proteolysis, and PERK through controlled translational regulation. These transcriptional responses mainly assist in the ability of protein folding in the RE. Furthermore, PERK and IRE1 are capable of reducing the folding load in the ER by blocking translation and degradation of mRNAs, respectively. In the proposed model, infection with vaccinia viruses (VACV) induces the activation of ATF6 and, simultaneously, the expression of ER chaperones and other responsive genes of the UPR pathway, which are primarily dependent on ATF6. The ATF6 branch is important for efficient viral replication of VACV. On the other hand, the absence of PERK benefits the production of the CEV and EEV virus infective forms. PERK is known to be inhibited by the expression of the viral protein K3, which has homology to eIF2a and functions as a pseudosubstrate for kinases such as PERK. Regardless of the increase in XBP1 expression levels, XBP1 mRNA processing is negatively modulated by VACV. The kinase (K) domain of IRE1 plays an important role in the productivity of VACVs. Positive and negative signals in red signal positive and negative modulation of pathways by VACVs.

There are three proteins residing in the endoplasmic reticulum that are responsible for sensing ER stress and are the main signalers of the UPR pathway: these are the protein kinase dependent on inositol (inositol-requiring transmembrane kinase and endonuclease, or IRE1), PKR-like ER-resident protein kinase (PERK), and ATF6 leucine zipper transcript (activating transcription factor 6) [30] (Figure 1). These molecules are kept inactive by binding to the main and most abundant ER chaperone, BiP/GRP78, through the luminal domain of these sensors [31,32]. The most conserved axis of the UPR pathway is signaled by IRE1α, a transmembrane glycoprotein of type I that uses a unique splicing mechanism that leads to the expression of genes related to protein folding and quality control. This sensor presents a ER luminal portion that is sensitive to misfolded polypeptides. Activation of this pathway occurs upon the binding of misfolded proteins to IRE1, resulting in a conformational change and activation of the endoribonuclease domain. Activation of this domain enables the post-transcriptional processing of the mRNA XBP-1 (X-box binding protein) transcription factor resulting in a different transcription factor of the b-ZIP family (leucine zipper and the basic region of the protein that is responsible for binding to DNA). This results in the expression of chaperones, modifying enzymes, and membrane remodeling [8,9,25,32,33,34,35].

Another UPR via sensor is called PERK, which, when activated, recognizes and phosphorylates the alpha subunit of the eukaryotic translation initiation factor (eIF2α), inactivating indirectly eIF2α and inhibiting the translation of mRNAs. Consequently, PERK assists in reducing the flow of protein synthesis and mitigating stress. Under stressful conditions, the reduction in eIF2α levels facilitates the translation of transcription factor 4 (ATF-4), which induces the transcription of important genes such as CHOP, GADD34, and ATF-3, related to resistance to oxidative stress, amino acid transport, and glutathione biosynthesis [9,36]. GADD34, via its subunit regulator, can dephosphorylate eIF2α, and, thus, restore normal conditions of proteins and convert ATF-4 translation to baseline levels. PERK’s ability to reverse conditions after episodes of stress is important to prevent damage caused by depletion of eIF2α [5].

ATF6 is a type II transmembrane protein residing in the ER, and whose main domain is in the ER lumen. When the accumulation of misfolded ATF-6 proteins occurs, it is transported through vesicles to the Golgi complex, where the sensor is cleaved by two proteases, site 1 (S1P) and site 2 (S2P), generating an amino-terminal domain of 50 kDa (NATF6/p50ATF6). This domain is then translocated to the nucleus, where it activates the transcription of UPR target genes, resulting in the synthesis of chaperones, and chaperone-modifying enzymes like CHOP [9,31].

### 2.2. UPR Pathway and Viral Infections

In the course of a productive infection, viruses induce host cells to produce large amounts of viral proteins, and unlike eukaryotes and prokaryotes, viruses lack chaperones such as heat shock proteins (HSPs) and rely mostly on host HSPs and other molecular chaperones for viral protein folding. In addition, many viral proteins undergo glycosylation and other post-translational modifications in the ER, overloading the organelle and causing activation of the UPR. Some consequences of UPR responses, such as the expression of chaperones and regulation of metabolism, may be useful for viral replication. However, block/attenuation of protein synthesis and apoptosis is not conducive to maximal viral replication. Understanding the mechanisms of interaction between viral infections and the UPR pathway provides valuable insights into the effects of viral infections on the UPR pathway and how viruses can benefit from this interaction. Indeed, all viruses that express glycoproteins, secreted proteins, or have a more complex replication cycle will interact with the pathway, either directly or indirectly. Moreover, a clear connection between UPR responses and vaccine efficacy has seldom been made. It is well established that UPR and both innate and adaptative immune responses are inherently intertwined [37]. However, how would a vaccine system based on intracellular expression of antigens affect UPR pathways and, ultimately, immune responses? Viral vectors such as adenoviruses and poxviruses drive intense—almost abusive—protein synthesis in the ER and that certainly affects cell homeostasis and triggers UPR responses. The same phenomenon may happen when other gene-delivery immunogenic strategies are used, including the disruptive mRNA vaccines that have contributed so intensively to controlling the COVID-19 pandemic. In both vectorial and nucleic acid-based vaccine strategies, the strength of the promoters driving expression of the foreign gene may have to be fine-tuned to avoid a decrease in cell translation, or even cell death, triggered by excessive stimulation of UPR sensors. That is something that remains to be further investigated. The interplay between UPR pathways and antigen production inside cells will be further discussed below.

Therefore, many viruses have evolved different mechanisms to selectively activate part of the UPR to relieve ER stress [38] (see Table 1).

Several studies have demonstrated viral ability to modulate UPR responses in mammal cells infected with viruses of the families *Arenaviridae*, *Asfarviridae*, *Peribunyaviridae*, *Caliciviridae*, *Coronaviridae*, *Flaviviridae*, *Hepadnaviridae*, *Orthoherpesviridae*, *Orthomyxoviridae*, *Paramyxoviridae*, *Parvoviridae*, *Picornaviridae*, *Sedoreoviridae*, *Retroviridae*, and *Togaviridae*. All can interfere with the signaling of at least one of the arms of the UPR pathway [39,40,41,42,43,44,45,46]. Nonetheless, how the UPR sensors recognize viral infection to activate the pathway is not yet fully understood. A study conducted by Sung et al. (2009) using the SARS coronavirus identified a SARS-CoV accessory protein (8ab protein) capable of binding directly to the luminal domain of ATF6. Ectopic expression of the 8ab protein in mammalian cells induces ATF6 proteolysis and translocation to the nucleus [47]. This finding suggests that perhaps viruses have evolved proteins to directly modulate the pathway UPR.

Among the *Orthoherpesviridae,* one of the most studied viral families in this context, several proteins capable of interfering with the UPR pathway have been identified. Viruses such as the Epstein Barr (EBV/*Lymphocryptovirus humangamma4*) infect B lymphocytes, professional secretory cells that need a fully functional ER and strongly depend on the UPR pathway [48]. One strategy used to ensure viral replication is to dose-activate the UPR pathway through the synthesis of a viral protein—the latent membrane protein 1 (LMP-1)—that mimics the CD40 receptor, induces the expression of NF-κB, and culminates in the activation of IRE1, PERK, and ATF6 [49]. Human herpesvirus type I (HHV-1/*Simplexvirus humanalpha1*) induces selective UPR activation through IRE1 but inhibits ATF6 sensor-dependent pathway in both epithelial and neuronal cells [49]. The cytomegalovirus (CMV), another member of the *Herpesviridae* family, has a different strategy from that used by EBV; CMVs have viral proteins capable of interacting with IRE1 and negatively regulating its activity; and the expression of one such protein (M50) is capable of inducing a reduction in the expression levels of this sensor [50]. Decreasing IRE1 expression by CMVs can prevent cellular responses that are likely detrimental to the replication of these viruses. The IRE1 pathway is also suppressed during infection of HeLa cells by HHV-1. XBP1 expression is reduced by the HHV-1 protein UL41, a highly specific endoribonuclease that leads to rapid degradation of HHV-1 mRNAs. HHV-1 and human herpesvirus 8 (HHV-8/*Rhadinovirus humangamma8)* modulate the activation of the PERK and IRE1 axes, mainly preventing the induction of pro-apoptotic pathways [51,52].

The African swine fever virus (ASFV), a member of the family *Asfarviridae,* and its K205R protein induces activation of the UPR pathway, autophagy, and stimulation of the NF-κB signaling pathway. The K205R protein triggers ER stress by activating key UPR components, specifically through the ATF6, IRE1, and PERK signaling pathways. Additionally, K205R inhibits the mTOR signaling pathway while promoting autophagy via the ULK1 kinase. Notably, K205R also facilitates the nuclear translocation of NF-κB (P65), thereby amplifying inflammatory responses. Inhibition of ER stress using a PERK inhibitor has been shown to reduce both autophagy and NF-κB activation induced by K205R, underscoring its crucial role in modulating cellular responses during ASFV infection [53].

Members of the *Flaviviridae* family, such as Dengue viruses (DENV/*Orthoflavivirus denguei*) also use different strategies to modulate the UPR. Studies have shown that different DENV serotypes modulate UPR differently. The four serotypes converge on the increased production of some molecules such as BiP/GRP78, GADD34, CHOP, XBP1, and eIF2α. However, they differ when evaluating the time of infection. In DENV-2 and DENV-4 infections, for instance, there is a decrease in the activation of these molecules within 48 h after infection (hpi), which are maintained for DENV-1 and DENV-3. The level of eIF2α phosphorylation is kept high for these last two serotypes, even after 48 hpi [54,55]. Another study found the induction of XBP1 processing by the non-structural protein NS2B in infection with DENV-2 [56]. GADD34 mRNA levels were up-regulated during the late phase of DENV infection. This molecule promotes eIF2α dephosphorylation and, therefore, functions as a negative feedback mechanism to trigger recovery from the blocking in protein translation. However, the inhibition of PERK in the early stages of infection, despite inducing repression of host translation, does not appear to affect viral protein levels [57]. Zika virus (ZIKV/*Orthoflavivirus zikaense*) has been shown to modulate the UPR pathway both in vitro and in vivo [58,59,60]. Gladwyn-Ng and collaborators (2018), using a ZIKV sample of Asian origin (H/PF/2013, GenBank: KJ776791.1), showed the occurrence of ER stress and activation of UPR both in postmortem cortical samples from human fetuses and in cultures of human neural stem cells infected with ZIKV, with a modest increase in IRE1-XBP1 activation and upregulation of PERK-ATF4, as well as an increase in production of some chaperones, such as calregulin and PDI. On the other hand, there was no observable activation of the ATF6 arm of the UPR pathway. In an in vivo model of infection, the restoration of cortical neurogenesis was observed in an experiment using PERK inhibitors, which decreased activation of apoptosis, or IRE1 inhibitors, which decreased virus replication and prevented the activation of PERK-ATF4. These data suggest a cross-regulation between these pathways. Tan et al. (2018), using another sample of Asian origin (Zika virus/SZ01/2016/China, GenBank: KU866423.2), identified increased transcription of several genes from the three arms of the UPR pathway: BIP, ATF6, phosphor-eIF2α, XBP1, ATF4, GADD34, CHOP, and processed EDEM-1 increased in vivo at 24 dpi and 48 dpi, using human neuronal cells and in mice deficient in receptors for IFN-I and IFN-II. In addition, they detected the translocation of XBP1 and ATF6 to the nucleus of infected cells in the brain of mice five days after infection, indicating activation of these pathways. Nonetheless, in different cellular models, different UPR modulation profiles were observed in cells infected with this ZIKV strain: BIP, phospho-IRE1, ATF4, and ATF6 increased significantly by 48 dpi. For both strains, there was an increase in phosphorylation of elF2α, XBP1 processing, and increased expression of GADD34 and CHOP.

The relationship between the UPR, NF-κB, and IFNs during viral infections is complex and essential for the host immune response. Infection with Porcine reproductive and respiratory syndrome virus (PRRSV) activates the UPR pathway, leading to an upregulation of type I interferon and IFN-stimulated genes. The activation of UPR induced the expression of protein kinase R (PKR), which is closely linked to a reduction in PRRSV replication. When cells were treated with tunicamycin, a UPR activator, or when PKR was overexpressed, early activation of the NF-κB signaling pathway and the interferon response occurred during PRRSV infection. This resulted in elevated production of type I interferons and pro-inflammatory cytokines, effectively inhibiting viral replication. Additionally, the PRRSV nonstructural protein nsp4 was found to downregulate PKR expression, potentially serving as a viral strategy to evade host antiviral defenses [61].

HCV causes endoplasmic reticulum stress, leading to activation of the UPR and subsequent production of interferons. However, HCV also has strategies to avoid this response, showing a dynamic interaction between the virus and host defense mechanisms. HCV activates the UPR pathway via its E1 and E2 glycoproteins; however, the virus appears to inhibit specific components of this response. HCV infection enhances the splicing of Xbp1 but does not robustly activate downstream targets, such as EDEM (ER degradation-enhancing alpha-mannosidase-like protein), which is crucial for the degradation of misfolded proteins. Additionally, in Ire1α-deficient cells (Ire1α being the sensor that activates Xbp1), higher levels of HCV glycoproteins were observed, likely due to reduced EDEM levels, impairing the degradation of viral proteins. This suggests that HCV suppresses the Ire1–Xbp1 signaling axis to facilitate the production of its envelope proteins. Moreover, this suppression may also help reduce the production of pro-inflammatory cytokines, which are typically upregulated through Xbp1 activation in response to viral pattern recognition [62].

Our group has also evaluated the effect of infection by Asian and African lineages of ZIKV on the activation of components of the UPR pathway in T98G human neuronal glioblastoma cells. The ATF6 arm of the UPR pathway is not activated in the infection by the Asian and African strains, which suggests negative modulation during the infection. Asian ZIKV inhibits the IRE1–XBP1 pathway at the early stages of infection by blocking the processing of XBP1. Both Asian ZIKV and African ZIKV modulate the activation of PERK–ATF4 in the late phases of infection, whereas the CHOP gene expression is down-regulated at the beginning of infection with the Asian strain. The African strain, on the other hand, seem to induce high levels of this protein expression. Generally speaking, Asian ZIKV inhibits UPR activation by up to 48hpi and activates it forcefully after 72hpi. The African strain seems to behave differently, and activate UPR pathways early during infection.

The Myxoma virus (MYXV/*Leporipoxvirus myxoma*), a member of the *Poxviridae* family, is also capable of modulating the UPR pathway. MYXV inhibits CHOP expression as well as ATF4, negatively affects XBP1 processing, and triggers ATF6 cleavage [63]. Jindal and Young (1992) showed that during infection by the Vaccinia virus, proteins of the Hsp70 family play a crucial role in the folding of viral proteins, and high levels of expression of Hsp70 proteins are detected, but not members of the Hsp90 and Hsp60 family [64]. The replicative success of the *Poxviridae* is partially dependent on viruses’ ability to block, evade, or subvert essential elements of the host’s responses to infection. Poxviruses have a double strand of DNA and encode about 200 proteins. The multiplication cycle of poxviruses is closely related to the ER, as the viral factories produced during poxvirus replication are surrounded by ER membranes [65,66]. Our research group has recently evaluated the effects of infection by different VACV strains on the cell responses to unfolded or misfolded proteins (Figure 1). In these studies, different cell lines were infected with cytopathic and virulent VACV-WR and non-virulent (modified vaccinia Ankara (MVA) virus. It was observed that both WR and MVA are capable of negatively modulating IRE1-mediated XBP1 splicing. On the other hand, both VACV strains induce nuclear translocation of ATF6, in addition to significantly inducing its transcriptional activity. Using cells deficient for the PERK sensor, we observed that VACV-WR multiplies efficiently even in the absence of this UPR pathway axis. However, when evaluating the multiplication of VACV-WR in cells deficient for the ATF6 sensor, we noted that these viruses have lower productivity when compared to infection in wild-type cells [67]. Moreover, we have also UPR modulation by two zoonotic VACV strains isolated from rural areas in Brazil. These strains have different virulence profiles: Vaccinia virus Guarani P1 (GP1V) is virulent in mice and belongs to group 2 of zoonotic VACVs; Passatempo virus (PSTV) is shown to be avirulent upon mice infection and belongs to group 1. The ATF6 sensor appears to be important for the replication of both zoonotic strains. We have also observed that the kinase domain of the XBP1 protein plays an important role in plaque phenotype and impacts viral productivity, in contrast to the RNase domain, which appears to be irrelevant for pathogenicity or viral productivity. The absence of PERK seems to increase infection by both the CEV and EEV infective forms of the zoonotic VACVs [68].

**Table 1:** Virus’ strategies to counteract UPR responses.

Virus *	Viral Protein	Affected UPR Pathway Receptor	Reference
SARS-CoV (*Betacoronavirus pandemicum*)	8ab protein	ATF6 activation	[47]
Epstein Barr (*Lymphocryptovirus humangamma4)*	LMP-1	Activation of PERK, IRE1, and ATF6	[48,49]
Murine cytomegalovirus (*Muromegalovirus muridbeta1*)	M50	Negatively regulates IREI	[50]
Human cytomegalovirus (*Cytomegalovirus humanbeta5*)	UL50	Negatively regulates IREI	[50]
Human herpesvirus 1 (*Simplexvirus humanalpha1*)	UL41	Reduced XBP1 expression	[52]
Dengue virus type 2 (*Orthoflavivirus denguei*)	NS2B	Induction of XBP1 processing	[56]
Adeno-associated virus vectors based on serotype (AAV) 2	Not described	Activation of IRE1 and PERK	[39]
African swine fever virus	K205R	Activation of ATF6, IREI, and PERK	[53]
Zika virus (*Orthoflavivirus zikaense*)	Not described	Activation of IRE1–XBP1 and PERK–ATF4	[58,59,60]
Myxoma virus (*Leporipoxvirus myxoma*)	Not described	Activation of ATF6 and inhibition of CHOP, ATF4, and XBP1	[63]
Vaccinia virus (*Orthopoxvirus vaccínia*)	Not described	ATF6 activation, XBP1 inhibition	[67,68]

* Current ICTV species denominations are indicated in parentheses.

### 2.3. UPR and Future Perspectives: Optimization of Heterologous Expression Using Viral Vectors and Vaccine Production

Heterologous protein expression utilizing viral vectors is a widely adopted and effective technique in biotechnology, enabling the efficient production of recombinant proteins. The optimization of this process is significantly influenced by factors such as codon usage and the UPR. Activation of the UPR enhances the folding capacity of the ER, thereby improving the yield of correctly folded recombinant proteins. This is particularly relevant when viral vectors are employed, as they may induce cellular stress responses due to elevated levels of protein synthesis. For instance, alphaviruses such as the Semliki Forest virus effectively inhibit host protein synthesis while supporting high levels of viral and heterologous protein production [69]. In the context of gene therapy, optimizing transgene codon usage and understanding the role of UPR in protein production can lead to improved therapeutic outcomes. Modulating UPR signaling may help reduce cytotoxic effects associated with high-level expression of therapeutic proteins [70,71].

The development of glycoprotein mRNA vaccines has gained considerable attention, particularly in response to the COVID-19 pandemic. These vaccines utilize lipid nanoparticles (LNPs) to deliver modified mRNA encoding viral glycoproteins, which are essential for inducing immune responses. The interaction between these vaccines and the UPR pathway is critical for understanding their efficacy and safety. From a vaccine design perspective, comprehending how glycoprotein mRNA vaccines engage with the UPR can inform strategies for enhancing vaccine effectiveness. For example, optimizing codon usage within mRNA sequences could alleviate ER stress and improve protein folding efficiency, potentially leading to better immunogenic outcomes. Given that UPR activation serves a dual role—beneficial for immune activation but harmful if dysregulated—monitoring UPR-related biomarkers may be essential for evaluating vaccine safety [70].

Additionally, the UPR is closely associated with the production of pro-inflammatory cytokines, which play a pivotal role in amplifying immune responses. During viral infections or post-vaccination, UPR activation can lead to elevated levels of cytokines such as IL-6 and TNF-α, which are crucial for mounting an effective immune response. However, while UPR activation can enhance immunity, excessive or prolonged activation may result in apoptosis or chronic inflammation. Achieving a balance in UPR activation is, therefore, critical for ensuring effective vaccine-induced immunity without triggering adverse effects [72,73].

## 3. Conclusions

Infection by viruses represent a challenge to the ER capability of protein production inside infected cells. Virus infections are prone to overload the ER, as viral strategies usually reside in producing massive amounts of proteins to assemble the virus progeny and leave the cell for another round of infection. UPR responses have evolved as a cellular mechanism to manage the production of misfolded proteins under physiological conditions. The UPR pathway aims to regulate protein synthesis, focusing on the normalization of folding and cell viability. However, upon cell stress and protein synthesis overload caused by viruses, UPR signals are directed to cell death by apoptosis as a way to cope with infection. Therefore, is not a surprise that viruses have evolved to counteract the death signals induced by the UPR pathways and even take advantage of the pro-life UPR signaling to maximize progeny generation. Although UPR modulation by viruses have been described for a relatively small group, is quite probable that most viruses have evolved interplay mechanisms to deal with ER stress signaling.
